# Molecular Discovery of Filarial Nematode DNA in an Endangered Wild Pinniped (Galapagos Sea Lion, *Zalophus wollebaeki*)

**DOI:** 10.1002/ece3.70596

**Published:** 2024-11-25

**Authors:** Isabella G. Livingston, Taylor M. Gregory, Eleanor C. Hawkins, Ashley Cave, Andrea Loyola, Shelly L. Vaden, Diane Deresienski, Marjorie Riofrío‐Lazo, Gregory A. Lewbart, Diego Páez‐Rosas, Matthew Breen

**Affiliations:** ^1^ Department of Molecular Biomedical Sciences, College of Veterinary Medicine North Carolina State University Raleigh North Carolina USA; ^2^ Department of Clinical Sciences, College of Veterinary Medicine North Carolina State University Raleigh North Carolina USA; ^3^ Greensboro Science Center Greensboro North Carolina USA; ^4^ Departamento de Ecosistemas Dirección Parque Nacional Galapagos Isla Santa Cruz Islas Galapagos Ecuador; ^5^ Galapagos Science Center USFQ & UNC‐Chapel Hill Isla San Cristóbal Islas Galapagos Ecuador; ^6^ Colegio de Ciencias Biológicas y Ambientales Universidad san Francisco de Quito (USFQ) Islas San Cristóbal Islas Galapagos Ecuador; ^7^ Dirección Parque Nacional Galapagos Oficina Técnica San Cristóbal Isla San Cristóbal Islas Galapagos Ecuador; ^8^ Comparative Medicine Institute North Carolina State University Raleigh North Carolina USA; ^9^ Center for Human Health and the Environment North Carolina State University Raleigh North Carolina USA

**Keywords:** endangered species, Galapagos, heartworm, introduced species, pathogen detection, sea lion

## Abstract

Rapidly changing environments are contributing to the spread of non‐native species and their associated pathogens into new and vulnerable ecosystems, such as the Galapagos archipelago. These pathogens represent a significant threat to emblematic species. The Galapagos sea lion ( *Zalophus wollebaeki* ) (GSL) is an endangered and endemic pinniped that is increasingly at risk of acquiring infectious diseases due to interactions with introduced companion animals. Previously, we reported the first detection of antigens from *Dirofilaria immitis*, the parasite that causes canine heartworm disease, in the GSL. To investigate further, we developed a multifilarial PCR assay and successfully detected DNA from *D. immitis* and the closely related *Dirofilaria repens* in 10.7% of our sample cohort of juvenile GSLs. This assay, based on a conserved region in the filarial 28S gene, can be used in conjunction with restriction endonuclease digestion or Sanger sequencing to identify the species of the causative nematode. Our method proved effective without nonspecific amplification in a wide host range, and highly sensitive, detecting as little as one parasite. Further, this assay can be used in cases of immature, low‐worm burden, or all‐male infections. Our molecular approach offers a sensitive and specific method for detecting filarial parasites in wild animals. Further investigations are necessary to confirm the pathology of filarial nematodes in the GSL and their prevalence in the general population. Our identification of Dirofilarial species in the GSL underscores the urgent need for measures to manage the risk of pathogen transmission from introduced species to native wildlife.

## Graphical abstract

Our research explores to use and optimization of molecular methods for the detection and identification of DNA from filarial nematodes in Galapagos sea lion whole blood samples. We developed an accessible workflow for the genus and species‐level identifications of filarial nematodes present in wildlife DNA samples, and used this assay to test 28 juvenile Galapagos sea lions.

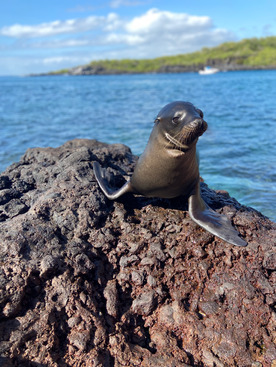

## 1 Introduction

The spread of non‐native species, including companion animals, into vulnerable ecosystems (Roy et al. 2023; Levy et al. 2008; Kaiser 2001; Padilla et al. 2018; Diaz et al. 2016) presents a significant threat to wildlife through competition, predation, and morbidity and mortality caused by introduced pathogens (Roy et al. 2023; Levy et al. 2008; Kaiser 2001; Padilla et al. 2018; Diaz et al. 2016). Non‐native species may host pathogens that do not naturally occur in the ecosystem and thus facilitate their establishment and potential spillover to native or endemic species. As such, non‐native species are a significant source of “pathogen pollution,” which can increase the risk of infection for wildlife and humans (Roy et al. 2017; Chinchioid et al. 2020).

The Galapagos archipelago, designated a UNESCO World Heritage Site (UNESCO World Heritage Convention), is renowned for its biodiversity and endemism, with species uniquely adapted to their respective habitats (Adsersen et al. 2002; Urquía et al. 2019). Due to the geographical isolation of the islands and the short history of human activity, wildlife species have few natural competitors and/or predators, increasing their vulnerability to disturbances (Cayot, Campbell, and Carrión 2021; Loope, Hamann, and Stone 1988; Phillips, Wiedenfeld, and Snell 2012). Furthermore, as oceanic island species, native wildlife likely evolved without significant pathogen exposure (Padilla et al. 2018). Thus, non‐native species, including companion animals and introduced pathogens, pose a direct threat to the wildlife of the Galapagos islands and contribute to the rapid degradation of Galapagos ecosystems (Urquía et al. 2019; Galapagos Conservation Trust; Verónica Toral‐Granda et al. 2017; Alava et al. 2023).

Over the past two centuries, approximately 1500 non‐native species have been introduced to the archipelago, serving various purposes including agriculture, food, and companionship (Padilla et al. 2018; Galapagos Conservation Trust). From 2014 to 2018, the island of Santa Cruz saw a 55% increase in dog populations, indicating a rise in domestic animal populations alongside human populations (Hernandez et al. 2020). The increasing domestic animal populations heighten the risk of disease transmission to endemic species, which poses a major conservation concern (Sarzosa et al. 2021; Kilpatrick et al. 2006). As companion animal populations continue to grow across the archipelago, the spread of infectious organisms to native wildlife will likely increase (Ruiz‐Saenz et al. 2023; Culda et al. 2022). Therefore, there is an urgent need to mitigate the risk of transmission of pathogens from companion species.

**FIGURE 1:**
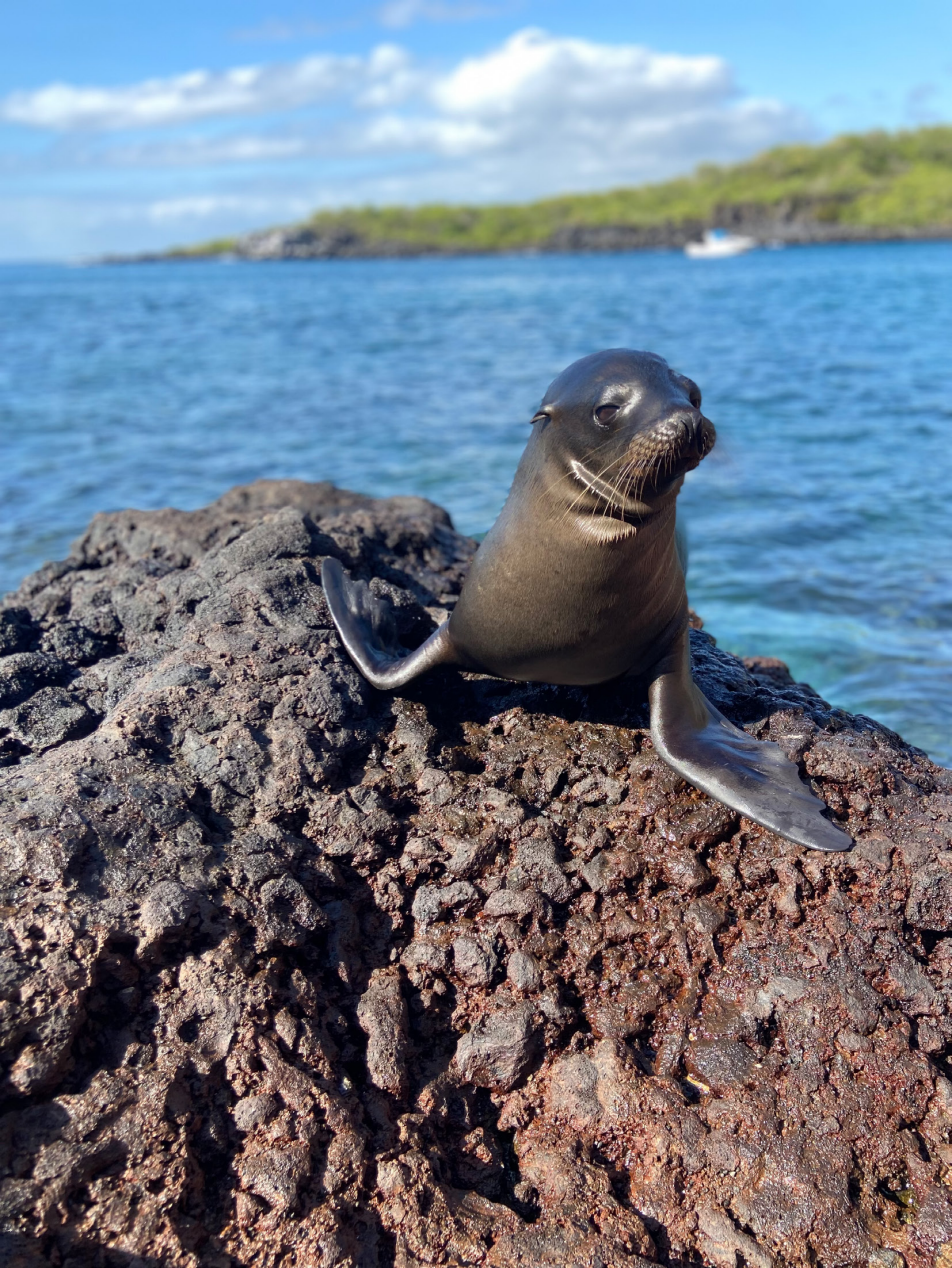
Juvenile Galapagos sea lion ( *Zalophus wollebaeki* ) on the beach at San Cristobal Island. Photo taken by Emily Schlake during May 2022 sample collection trip.

The Galapagos sea lion ( *Zalophus wollebaeki* ) (GSL) (Figure 1) is one of the species most impacted by the presence of non‐native taxa. This endemic pinniped has been identified by the Galapagos National Park Directorate as a sentinel species of ecosystem health with high conservation priority (Alava et al. 2011). However, their populations have declined by more than 50% in the last 40 years (Ruiz‐Saenz et al. 2023; Denkinger et al. 2017; Trillmich 2015; Páez‐Rosas et al. 2020). The GSL is sensitive to environmental fluctuations, such as El Niño events, which create periods of low productivity in the marine environment (Páez‐Rosas et al. 2020; Trillmich and Dellinger 1991). Such events cause increased nutritional stress and mortality rates in this species (Trillmich 2015). Thus, the stress that El Niño places on the GSL immune system may allow infectious agents with ordinarily low pathogenicity to become life‐threatening (Gregory et al. 2023). As a result, the International Union for Conservation of Nature (IUCN) cites introduced species as a critical threat to GSL survival and contributor to population declines (Culda et al. 2022; Trillmich 2015). Pinniped species are susceptible to pathogens that routinely infect domestic dogs, including canine distemper virus (CDV), *Mycoplasma* spp., leptospirosis, and parasitic nematodes such as *Dirofilaria immitis* (Culda et al. 2022; Katz et al. 2022; Alho et al. 2017). Antibodies against the organisms that cause CDV, *Mycoplasma* infections, and leptospirosis have been reported in the GSL (Culda et al. 2022; Denkinger et al. 2017), and *D. immitis* has been reported in other pinnipeds including the hooded seal (*Cystophora cristate*), common seals ( *Phoca vitulina* ), African fur seals (*Arctocephhalus pusillus*), and the California sea lion ( *Zalophus californianus* ) (Culda et al. 2022; Alho et al. 2017; Diakou, Deak, and Veronesi 2023).

In our previous study, we reported the detection of heartworm antigens in two out of 28 GSLs sampled at the El Malecon rookery on San Cristobal Island. This rookery is home to the largest population of GSLs in the archipelago and is in proximity to urban areas and companion animals (Culda et al. 2022; Gregory et al. 2023; Páez‐Rosas and Guevara 2017; Páez‐Rosas et al. 2021). Concurrently, we reported the morphological and molecular identification of 20 adult worms (*D. immitis*) recovered from the right ventricle of a deceased adult GSL from Santa Cruz. This represented the first documentation of *D. immitis* infections in the GSL (Gregory et al. 2023).

*Dirofilaria immitis* is a filarial nematode that causes cardiopulmonary dirofilariasis in canines, felines, wild mammals, and in rare cases, humans (Alho et al. 2017; Oh, Kim, and Sung 2017; Kronefeld et al. 2014; Fu et al. 2014; Dantas‐Torres et al. 2023; Nelson et al. 2018; Robinson and Robinson 2016; Gomes‐de‐Sá et al. 2022; Fontes‐Sousa et al. 2019; Nuchprayoon et al. 2005; McCall et al. 2008; Pietikäinen et al. 2017; Ferreira et al. 2017). Mosquitoes are obligate intermediate hosts of *D. immitis*. They ingest microfilaria, or first stage larvae (L1), during a blood meal from a host infected with adult *D. immitis* . The microfilaria develops into infective third stage (L3) larvae and migrate through the body cavity to the mosquito head and mouthparts (Nelson et al. 2018; McCall et al. 2008; Noack et al. 2021). Once in the mouthparts, they are subsequently deposited in the mosquito hemolymph produced when the mosquito takes a blood meal, and enter the hole left in the new blood host when the mosquito removes its stylet (Nelson et al. 2018; McCall et al. 2008). The L3 larvae then penetrate and mature in host tissues, at which point they migrate to the right heart and pulmonary arteries (Oh, Kim, and Sung 2017; Dantas‐Torres et al. 2023; Nelson et al. 2018; McCall et al. 2008). Heartworm disease in dogs primarily manifests from induced inflammation and hyperplasia of the vascular lining of the pulmonary arteries. This condition leads to reduced blood flow and pulmonary hypertension, with the potential for further obstruction by the worms themselves or emboli from deceased worm fragments. Coughing and impaired oxygenation during physical activity are prevalent clinical signs, and infection may be fatal if the worm burden is significant or if it is left untreated (Gregory et al. 2023; Laidoudi et al. 2020).

Of the over 3500 species of mosquito (Hawkes and Hopkins 2022), only three are found in the archipelago. These species are *Aedes taeniorhynchus* , *Culex quinquefasciatus* , and *Aedes aegypti* (family: Culicidae) (Culda et al. 2022; Asigau et al. 2018; Bataille et al. 2009), which are of significant medical importance due to their ability to transmit pathogens, including *D. immitis* , to wildlife (Asigau et al. 2018).

As pinnipeds, GSLs have an amphibious lifestyle and divide their time between aquatic environments and terrestrial habitats. On land, they are vulnerable to bites from mosquitoes and other disease vectors (Alho et al. 2017; Keroack et al. 2018). The presence of domestic dogs and identifications of *D. immitis* in these populations on the beaches of the human‐inhabited islands of the Galapagos suggests that infection by *D. immitis* poses a serious potential threat to the GSL (Culda et al. 2022; Gregory et al. 2023; Alho et al. 2017).

Current knowledge of the prevalence and risk of disease from *D. immitis* in pinnipeds is limited, as only a few cases of infection have been described thus far, and apart from our previous report, have been primarily restricted to captive individuals (Keroack et al. 2018; Krucik, Van Bonn, and Johnson 2016). Adult *D. immitis* nematodes have been found in the right ventricle of the heart, pulmonary arteries, vena cavae, portal vein, and the pericardial sac during pinniped necropsies (Alho et al. 2017). Clinical signs of infection, such as cardiopulmonary impairment, coughing, and labored breathing, have been documented in the California sea lion ( *Z. californianus* ) (Culda et al. 2022; Alho et al. 2017), the closest living relative of the GSL.

Though *D. immitis* is the most notable filarial species, other related species are relevant in veterinary medicine. *Dirofilaria repens* is a parasite of subcutaneous tissues in carnivores and is the leading cause of dirofilariasis in humans (Laidoudi et al. 2020; Gioia et al. 2010; Giannelli et al. 2013). Both species are prevalent in mosquito‐populated regions and are considered to have high vector‐borne zoonotic potential (Gomes‐de‐Sá et al. 2022; Fontes‐Sousa et al. 2019; Noack et al. 2021; Gioia et al. 2010; Latrofa, Dantas‐Torres, et al. 2012; Simón et al. 2005, 2012; Esteban‐Mendoza et al. 2020). Studies have indicated an increase in the incidence of *Dirofilaria* infections across tropical, subtropical, and temperate regions, as well as expansion into previously unaffected regions (Oh, Kim, and Sung 2017; Gioia et al. 2010). The proliferation of these infections has been postulated to correlate with climatic alterations, specifically the rising temperatures that facilitate mosquito reproduction and provide suitable conditions for infective larvae development (Kronefeld et al. 2014; Dantas‐Torres et al. 2023; Latrofa, Dantas‐Torres, et al. 2012; Morchón et al. 2012).

Species of the closely related genus *Acanthocheilonema* are routinely identified during necropsies of pinniped species. *Acanthocheilonema spirocauda* commonly infects phocids (earless seals such as the harbor seal and Hawaiian monk seal), while otariids (eared seals such as fur seals and sea lions) are infected by *A. odendhali* (Laidoudi et al. 2020). In contrast to *Dirofilaria* species, *A. odendhali* is considered nonpathogenic in otariids and has not been reported in the Galapagos (Krucik, Van Bonn, and Johnson 2016).

Methods for the fast and accurate identification of causative nematode species are essential to better understand the impact of these infections on the GSL and to identify epidemiologic factors that could be leveraged to minimize transmission from reservoir species such as the dog (Gioia et al. 2010). Such tools would also be useful in other settings globally where filarial worms are of cause for concern. Current methods routinely used for the antemortem diagnosis of *D. immitis* infections in dogs include the microscopic identification of microfilariae in the host's blood and antigen tests that detect proteins produced by adult female worms (Starkey et al. 2017). Microscopy methods necessitate expert differentiation of closely related and morphologically similar circulating microfilariae (L1) (Oh, Kim, and Sung 2017; Nuchprayoon et al. 2005; Laidoudi et al. 2020; Gioia et al. 2010). This can be challenging in areas where different filarial species co‐exist (Gioia et al. 2010; Pérez‐Ramírez et al. 2023). Antigen detection targets proteins released by the reproductive tract of female adult worms, which may yield false‐negative results within the first 5–8 months of infection, and in cases of low‐worm burden, all‐male infections, and/or if there is antibody–antigen binding (Oh, Kim, and Sung 2017; Dantas‐Torres et al. 2023; Starkey et al. 2017; Venco et al. 2017). Commercial antigen test kits for *D. immitis* infection have been documented to cross‐react with other filarial and non‐filarial nematodes, leading to potential misinterpretations (Laidoudi et al. 2020) and false classification of *D. immitis* infection. Furthermore, these test kits are designed specifically for canine and feline blood samples and are likely to perform poorly or lose sensitivity when used with pinniped samples, resulting in underestimates of the prevalence of the disease in their populations (Alho et al. 2017).

Molecular‐based detection techniques have the potential to facilitate the accurate identification of *D. immitis* and other filarial worms, enhancing the ability to monitor infections in wildlife populations (Oh, Kim, and Sung 2017; Gioia et al. 2010). Previously, we developed a PCR assay for the detection of *D. immitis* that can detect heartworm DNA even in cases of juvenile, all‐male, and low‐worm burden infections (Gregory et al. 2023). However, this test was limited to the detection of *D. immitis* DNA and could not detect other closely related species (Latrofa, Weigl, et al. 2012). There are limited methods that offer simple and unambiguous procedures for heartworm in wildlife species (Gioia et al. 2010).

There is a need to establish reliable and cost‐effective measures for monitoring and mitigating the impact of introduced pathogens on the biodiversity of one of the world's most pristine ecosystems. In this study, we have expanded upon our initial protocol for detecting *D. immitis* using PCR in GSL blood. The optimized PCR assay can be used to distinguish between at least four filarial species relevant to the Galapagos and the GSL, with minimal equipment. Species identification can be done with a 156 base pair amplicon, which is especially useful in cases of degraded or low‐quality DNA samples. With a higher sensitivity and specificity than commercially available antigen tests, this assay aids in the detection of heartworm infections and offers an accessible tool for monitoring the prevalence of filarial species in GSLs.

## 2 Methods

### 2.1 Sample Collection

Twenty‐eight juvenile GSLs were captured with the aid of the Galapagos National Park Directorate personnel. Details of our sample collection are described in Gregory et al. (2023). Whole blood was collected, and the samples were transferred under the ethical review and approval granted by the Ministry of the Environment of Ecuador under the Framework Contract for Access to Genetic Resources MAE‐DNB‐CM‐2016‐0041 and the research permit DPNG‐PC‐19‐23. All fieldwork was carried out following the protocols of ethics and animal handling approved by the San Francisco de Quito University. Whole blood was kept at −80°C until processed.

### 2.2 DNA Extraction

All surfaces were disinfected with 70% ethanol prior to any laboratory procedures. Proper PPE was worn at all points during this work. Two positive controls were generated by extracting DNA from *D. immitis* microfilariae in infected canine peripheral mononucleated cells (PBMC) and bone marrow specimens. Canine blood and bone marrow were provided by the NC State College of Veterinary Medicine Clinical Pathology Laboratory. DNA was extracted from both positive controls as follows: each control specimen was centrifuged at 96 g for 10 min. After aspirating the supernatant, the pellets were resuspended in 500 μL of low TE buffer (10 mM Tris–HCL pH 8 0.1 mM EDTA) and centrifuged at 2085 g for 10 min. The supernatant was discarded, and the pellets were processed for DNA extraction using a Maxwell RSC Cell DNA Kit according to the manufacturer's protocol (Promega, Madison, WI, USA). DNA previously extracted from canine whole blood samples negative for heartworm was used as a negative control. Separately, 500 μL of whole blood (collected in EDTA, ethylenediaminetetraacetic acid) from each GSL was processed for DNA extraction using the Promega Maxwell RSC Whole Blood DNA Extraction Kit (Promega, Madison, WI, USA). Extracted genomic DNA was quantified using a Quantus Fluorometer (Promega, Madison, WI, USA), and DNA integrity was assessed by spectrophotometry and routine agarose gel electrophoresis.

### 2.3 PCR Primer Design and Optimization

Using sequence data accessible in GenBank for representatives of target nematode families, including four species of filarial nematodes ( *D. immitis* , *D. repens* , *A. odendhali*, and *A. viteae*), the 28S subunit of the large subunit rRNA gene was selected for PCR primer design for the multifilarial assay. The 28S gene has approximately 150 tandem repeats in the filarial nematode genome, improving PCR detectability (Laidoudi et al. 2020; Bik et al. 2013). Representative FASTA sequences for the 28S rRNA gene of all four available filarial species were downloaded from NCBI and aligned using Benchling software (Benchling Biology Software) ( *D. immitis* Accession: NP_954717, *D. repens* Accession: KP760376.1, *A. odendhali* Accession: KP760358.1, *A. viteae* Accession: KP760359.1). Two amplicons (156 and 295 bp) were selected for primer design using IDT PrimerQuest (Integrated DNA Technologies, Coralville, IA, USA). The regions were selected based on DNA sequence conservation across all considered filarial species at the beginning and end of each amplicon, with species‐specific variability in the middle of the amplicon (Appendix S1).

Primer sequence specificity and secondary structural properties were evaluated using the BLASTN tool accessible via the NCBI website. The physiochemical characteristics of each primer set were analyzed using the free online tool Multiple Primer Analyzer (ThermoFisher, Waltham, MA, USA). Primer sequences were cross‐referenced against DNA databases of various taxonomic groups to assess specificity (metazoans (taxid:33208), vertebrates (taxid:7742), bacteria (taxid:2), Canidae (taxid:9608), and pinnipeds (taxid:9703)). Each candidate primer sequence was assessed for predicted specificity via *in silico* PCR analysis using whole genome sequences of various potential host species as templates, including gray wolf ( *Canis lupus* ), rabbit ( *Oryctolagus cuniculus* ), deer ( *Odocoileus virginianus* ), orangutan ( *Pongo abelii* ), red wolf ( *Canis rufus* ), raccoon ( *Procyon lotor* ), pig ( *Sus domesticus* ), ferret ( *Mustela putorius furo* ), and cow ( *Bos taurus* ). Upon receipt, primers were resuspended in a low TE buffer to a final concentration of 100 μM from which working dilutions of 10 μM were generated.

**FIGURE 2:**
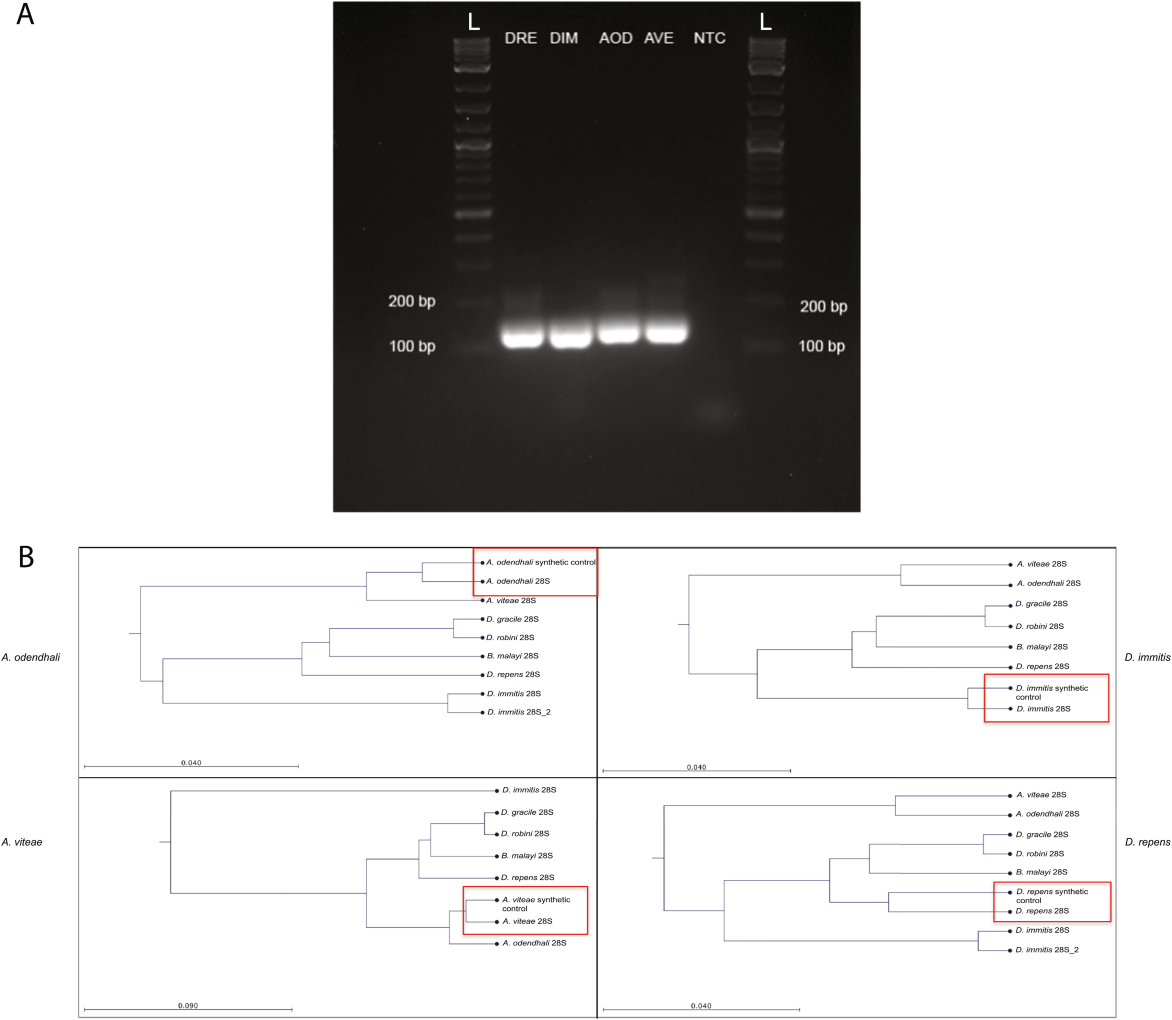
Multifilarial PCR and phylogenetic tree reconstruction of filarial nematode control specimens. The results of this assessment showed a successful PCR and taxonomic identification of the controls. (A) Gel Electrophoresis results for multifilarial PCR amplicons from nematode controls. All amplicons produced a band consistent with the predicted amplicon product for their respective species 28S rRNA sequence. From left to right: *Dirofilaria repens* (DRE), *Dirofilaria immitis* (DIM), *Acanthocheilonema odendhali* (AOD), *Acanthocheilonema viteae* (AVE), no‐template control (NTC). (B) Phylogenetic tree construction from Sanger sequences returned from multifilarial PCR amplicons of four nematode controls. Red boxes show the correct species identification of each of the four controls used. Trees were constructed using the UPGMA Tree Construction method, Jukes‐Cantor nucleotide distance measure, and 10,000 bootstraps.

The two primer sets were tested on the following templates: (1) DNA from a GSL blood sample, (2) DNA from an adult *D. immitis* specimen, and (3) microfilaria (with dog) DNA from canine PBMCs. For amplification, PCRs were prepared on ice with the following reagents: 3.25 μL of HPEC water, 6.25 μL of GoTaq Colorless Master Mix (Promega, Madison, WI, USA), 1.0 μL each of the 10 μM forward and reverse primers, and 1.0 μL of template DNA (10–25 ng/μL). Water was used in place of template DNA for the no‐template control. All PCRs were prepared on ice. The reaction conditions are shown in Table 1, and the PCR products were visualized by electrophoresis through a 2% agarose gel. Synthetic controls (g‐blocks) were designed for *A. odendhali*, *A. viteae*, and *D. repens* using their published 28S rRNA genes. While other species (*A. caudispina*, *A. gracile* , *A. robini*, and *Brugia malayi*) shared the conserved primer sequences, synthetic controls designed for these species did not pass IDT's g‐block construction. Synthetic gene fragments were resuspended to a final concentration of 10 ng/μL and used as template DNA for testing with the selected primer set (Figure 2A). The PCR products for each of the controls were subjected to bidirectional Sanger sequencing at the NC State University Genomic Sciences Library and species identity was validated using NCBI BLAST (Bataille et al. 2009) and CLC Sequence Viewer for phylogenetic tree reconstruction (QIAGEN CLC Sequence Viewer 8) (Figure 2B).

**TABLE 1:** Final reaction conditions for the multifilarial PCR assay.

Multifilarial PCR cycling conditions			
Step	Temperature (°C)	Time (HH:MM:SS)	Cycles
Initial denaturation	95	00:06:00	1×
Denaturation	94	00:00:20	30×
Annealing	54	00:00:20	
Extension	72	00:01:00	
Final extension	72	00:05:00	1×
Hold	12	∞	

To test the optimal annealing temperature of the selected primer set (Table 2), the following template samples were selected for PCR: 100% host DNA (GSL), 75% host, 25% parasite (spiked into GSL DNA), 99% host, 1% parasite (spiked into GSL DNA), and 100% parasite from adult *D. immitis* . The reagents used were the same as those used for initial primer testing, and the annealing temperatures were tested across a thermal gradient: 53°C, 53.4°C, 54.2°C, 55.3°C, 56.7°C, and 57.8°C (not shown). All temperatures tested produced bands consistent with the predicted amplicon size, but 54°C produced the brightest and cleanest band.

**TABLE 2:** Final primers used in the multifilarial PCR assay.

Multifilarial PCR primers					
Primer name	Marker	Primer sequence (5′–3′)	*T*_M_ (°C)	GC%	Length (bp)
Multifilarial forward	28S rRNA	CAGTCCATAGAAGGTGCTAGAC	59.9	50	22
Multifilarial reverse	28S rRNA	CTCACGGTACTTGTTTGCTATC	60.7	45.5	22

*Note:* Melting temperatures were calculated using a modified nearest‐neighbor method (Breslauer, Frank, Blöcker, and Marky 1986) and may vary based on calculation methods.

### 2.4 Final Multifilarial PCR Reaction Conditions

**FIGURE 3:**
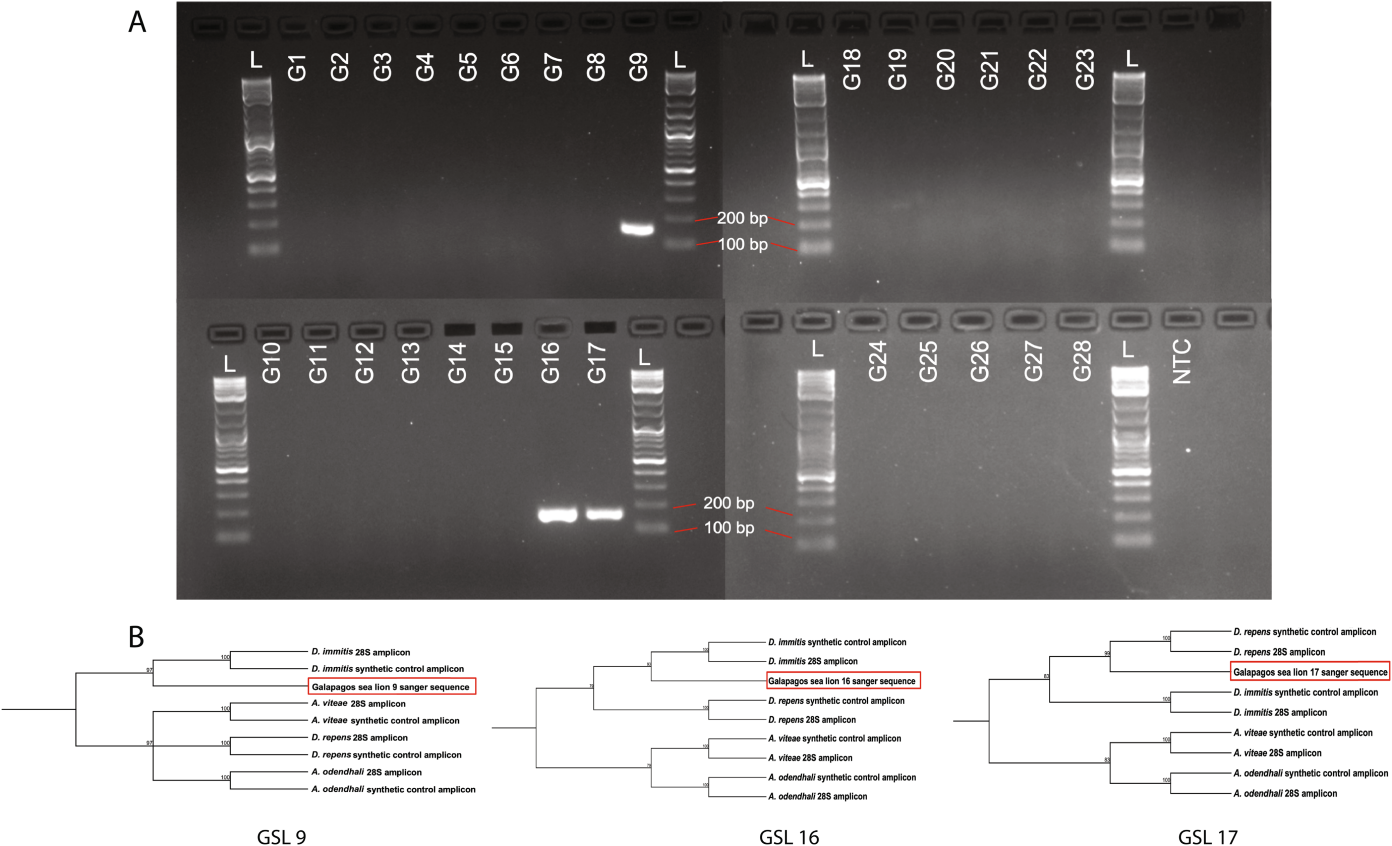
Multifilarial PCR of all GSL specimens and taxonomic identification of individuals positive for filarial nematode DNA. (A) Multifilarial PCR products of all 28 GSL samples and a no‐template control. Bands representing filarial DNA were seen in three samples (G9, G16, and G17), including the sea lion sample that was previously shown to have *Dirofilaria immitis* (G16). (B) Phylogenetic tree construction of amplicons produced from Sanger sequencing of Galapagos sea lion 9, 16, and 17 (all bordered in red). The resulting tree suggests the DNA sequenced from G9 and G16 was from *D. immitis* , and the DNA sequenced from G17 was from *Dirofilaria repens*, consistent with our BLAST analysis. Trees were constructed using the UPGMA Tree Construction method, Jukes‐Cantor nucleotide distance measure, and 10,000 bootstraps.

Genomic DNA isolated from EDTA blood samples of 28 GSLs was subjected to PCR based on the results of the optimization tests. All reactions were prepared on ice and run with a no‐template control, and synthetic DNA representing each of the four nematode species. The master mix was prepared with 3.25 μL of water, 1.0 μL of each 10 μM primer, and 6.25 μL of GoTaq Colorless MasterMix (Promega, Madison, WI, USA) per reaction. Template DNA (1 μL) from each filarial control was used for positive controls, representing 1.47 × 10^10^ copies of the template. The PCR reaction conditions are shown in Table 1 and amplicons were visualized on a 2% agarose gel (Figure 3A). All positive samples were submitted to the NC State University Genomic Sciences Laboratory for bidirectional Sanger Sequencing. The species identification of each amplicon sequence was performed using NCBI BLAST (Bataille et al. 2009). CLC Sequence Viewer (QIAGEN CLC Sequence Viewer 8) was used for basic phylogenetic tree reconstruction using the UPGMA tree construction method, Jukes‐Cantor nucleotide distance measure, and 10,000 bootstraps (Figure 3B).

### 2.5 Limit of Detection

**FIGURE 4:**
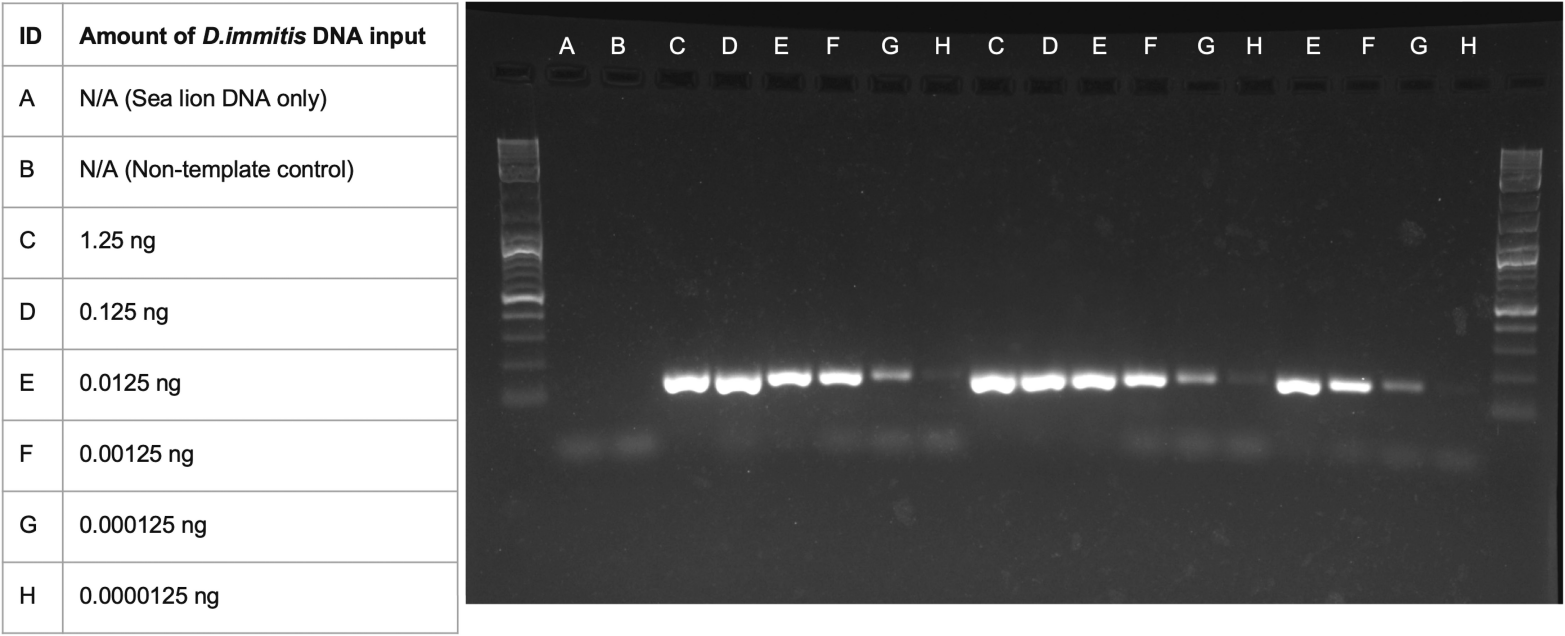
Multifilarial PCR assay limit of detection. DNA from a heartworm specimen was used to prepare serial dilutions and spiked into DNA from a GSL negative for heartworm. The limit of detection for this standard PCR is 1.25 × 10^−5^ ng of heartworm DNA (~3.67 × 10^4^ copies).

DNA isolated from an adult *D. immitis* specimen was used to prepare serial dilutions and spiked into DNA from a GSL negative for heartworm based on the results of the antigen test and multifilarial PCR assay. The concentration of heartworm DNA ranged from 1.25 ng/μL to 1.25 × 10^−4^ pg/μL (representing a range of 3.67 × 10^2^–3.67 × 10^9^ copies). The dilutions with the highest concentrations (1.25 ng/μL and 1.25 × 10^−1^ ng/μL) were tested in duplicate, and the dilutions with the lowest concentrations (1.25 × 10^−2^ ng/μL and 1.25 × 10^−4^ pg/μL) were tested in triplicate using the multifilarial standard PCR protocol. The results were visualized on a 2% agarose gel (Figure 4).

To test for limits of quantification, serial dilutions of filarial nematode DNA representing a range of 3.67–3.67 × 10^9^ copies were run on a qPCR assay. The samples were prepared using the SSoAdvanced Universal SYBR Green SuperMix (Bio‐Rad, Hercules, CA, USA) following the manufacturer's recommendations, and amplified with the multifilarial PCR primers. Each sample, including the no‐template control, was evaluated in triplicate with a temperature profile of 95°C for 6 min, 39 cycles of 95°C for 20 s, 55°C for 20 s, and 72°C for 1 min, and 72°C for 5 min. The melt curve analysis was from 65°C to 95°C at 0.5°C intervals. DNA from each filarial control species and simulated mixed infection samples were also run with this protocol to compare the differences in melt curve profiles. The quantitative results (*C*_q_) and melt curve data were analyzed for specificity and a standard curve equation was generated with the Bio‐Rad CFX Maestro software (Bio‐Rad, Hercules, CA, USA). The 28 GSL samples were then subjected to this qPCR protocol, and target specificity was validated via melt curve analysis and gel electrophoresis. The amount of nematode DNA in each sample and the copy number for the lower limit of quantification were calculated using the standard conversion equation for double‐stranded DNA.

### 2.6 Restriction Enzyme Analysis

**FIGURE 5:**
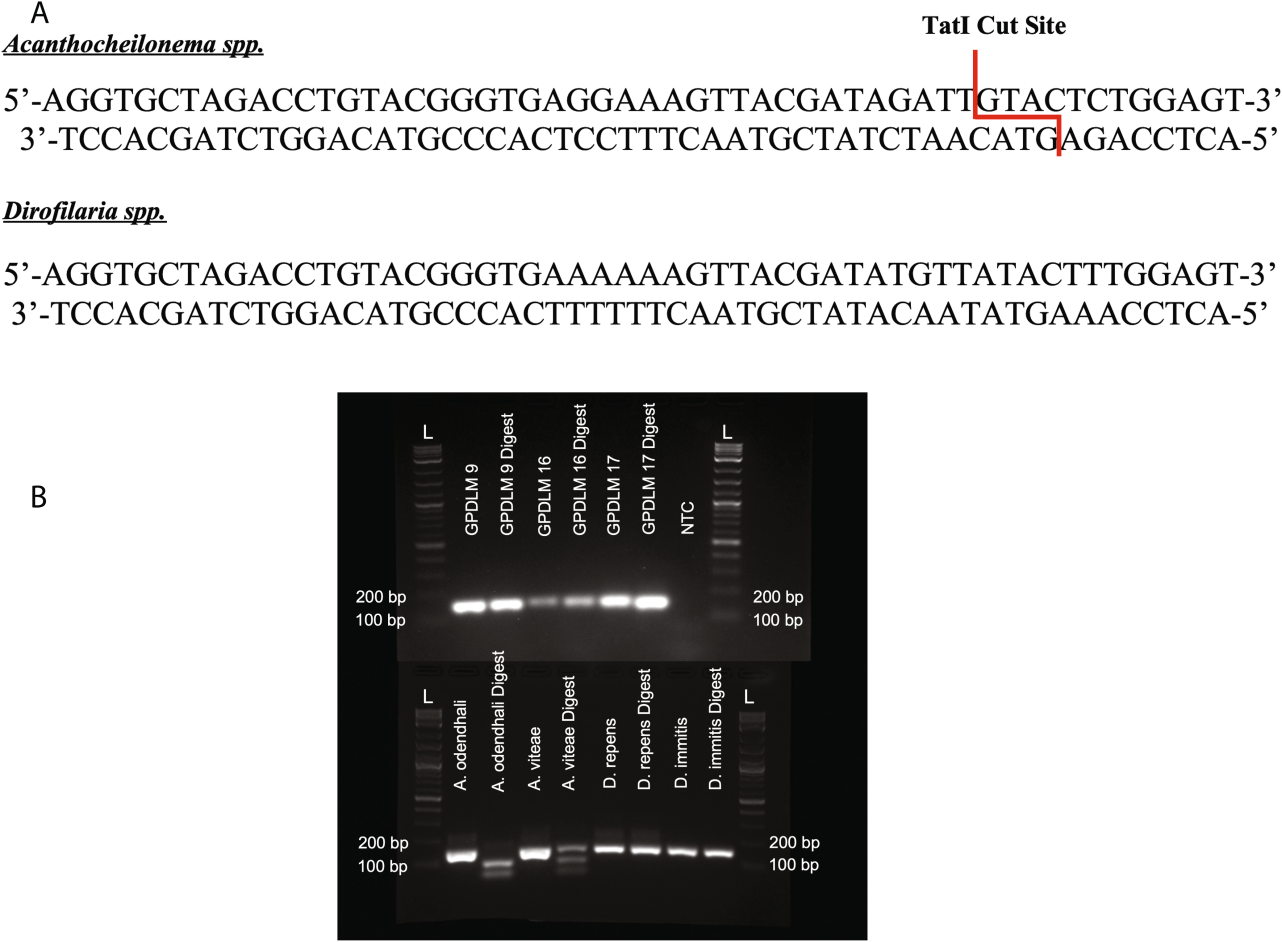
Restriction endonuclease digestion for genus‐level identification. (A) *Acanthocheilonema* spp. and *Dirofilaria* spp. multifilarial amplicon sequence with TatI restriction endonuclease cut site. The recognition sequence of the enzyme is specific to the *Acanthocheilonema* genus and will produce bands of approximately 56 and 100 basepairs. (B) Results of the restriction endonuclease digestion with the FastDigest TatI enzyme. Results are shown for the PCR amplicons and digested products for the 4 filarial species controls (*A. odendhali*, *A. viteae*, *D. repens* , and *D. immitis* ) and the three positive GSL individuals (G9, G16, and G17). Results of the digestion suggest that the species present in the GSL samples are from the *Dirofilaria* genus, consistent with our Sanger sequencing and phylogenetic analysis.

To identify alternative methods for species delineation, sequence data from the PCR amplicons of the synthetic controls representing four filarial species were uploaded to NEBcutter v3.0 (New England BioLabs, Ipswich, MA, USA) and used to identify restriction endonucleases. One enzyme, TatI, was selected as it was predicted to make digestions specific to the *Acanthocheilonema* species and was commercially available (Figure 5A). No enzymes that could distinguish between the *Dirofilaria* species were commercially available.

To identify the presence/absence of the TatI site, 10.0 μL of the multifilarial PCR products of three positive GSLs were added to a mixture comprised of 17.0 μL of nuclease‐free water, 2.0 μL of 10× FastDigest Green Buffer, and 1 μL of FastDigest TatI (Thermo Fisher Scientific, Waltham, MA, USA). Reactions containing amplicons of the four controls were also digested. Per the manufacturer's recommendations, the reactions were incubated at 65°C for 15 min and then 5 μL of each reaction was electrophoresed through a 2% agarose gel to identify the presence/absence of the TatI site (Figure 5B).

## 3 Results

The PCR primer sequences chosen for the multifilarial assay were conserved across many filarial species including at least four species from the subfamilies Onchocercinae and Dirofilariinae, and the genus *Mansonella*. PCR analysis of the genomic DNA of *D. immitis* and synthetic DNA sequences designed to represent the three filarial nematode controls (*A. odendhali*, *A. viteae*, *D. repens* ) produced single amplicons consistent with the predicted size (156–157 bp) for each species' 28S rRNA sequence. The BLAST analysis and phylogenetic tree construction of the Sanger sequencing results recovered the correct species identification in all four control samples (Figure 3B). Each control specimen shared the highest percent identity with their intended species (100% identity).

The multifilarial PCR assay was performed with genomic DNA isolated from a range of potential filarial host species. The results revealed that these primers can be used without producing nonspecific PCR products for all species tested: California sea lion, Galapagos sea lion, gray wolf, domestic dogs, rabbit, deer, orangutan, red wolf, raccoon, pig, ferret, and cow.

The limit of detection for the multifilarial PCR test was 1.25 × 10^−5^ ng (~3.67 × 10^4^ copies or 122 mf/μL) (Figure 4). The amplicon band, when visualized on a 2% gel, was faint but could be seen in each of the three replicates. The results from the qPCR assay showed that the lower limit of quantification, the lowest point on the standard curve at which all replicates of the standard curve were detected (Lappan et al. 2022), was 1.25 × 10^−7^ ng/μL (< 50 copies, or 1 mf/μL). The melt curve profile generated from testing of the positive controls and simulated mixed filarial samples ranged from 81.5°C to 84.5°C (Table 3), and the three GSLs positive for nematode DNA fell within this range. The remaining GSL samples either fell below this range or possessed dual T_M_ peaks that did not return viable Sanger sequences (Appendix S2). Using this standard curve, the quantities of nematode DNA present in specimens G9, G16, and G17 were 5.297 × 10^−5^ ng (1.55 × 10^5^ copies), 1.334 × 10^−3^ ng (3.91 × 10^6^ copies), and 1.063 × 10^−4^ ng (3.12 × 10^5^ copies), respectively. Based on the number of tandem repeats of the 28S gene in the filarial nematode genome, these copy numbers equate to approximately 517 mf/μL DNA, 13,052 mf/μL DNA, and 1040 mf/μL DNA, respectively.

**TABLE 3:** Melt curve profiles for each filarial species control‐tested and simulated samples of mixed infections.

Multifilarial qPCR melt curve profiles	
Species	Temperature (°C)
*Acanthocheilonema viteae*	84.0
*Acanthocheilonema odendhali*	84.5
*Dirofilaria repens*	83.0
*Dirofilaria immitis*	81.5–82.0
*D. immitis* + *D. repens*	82.5–83.0
*D. immitis* + *A. odendhali*	84.0
*D. repens* + *A. viteae*	83.5–84.0

*Note:* Profiles were used for assessment of GSL qPCR results.

**FIGURE 6:**
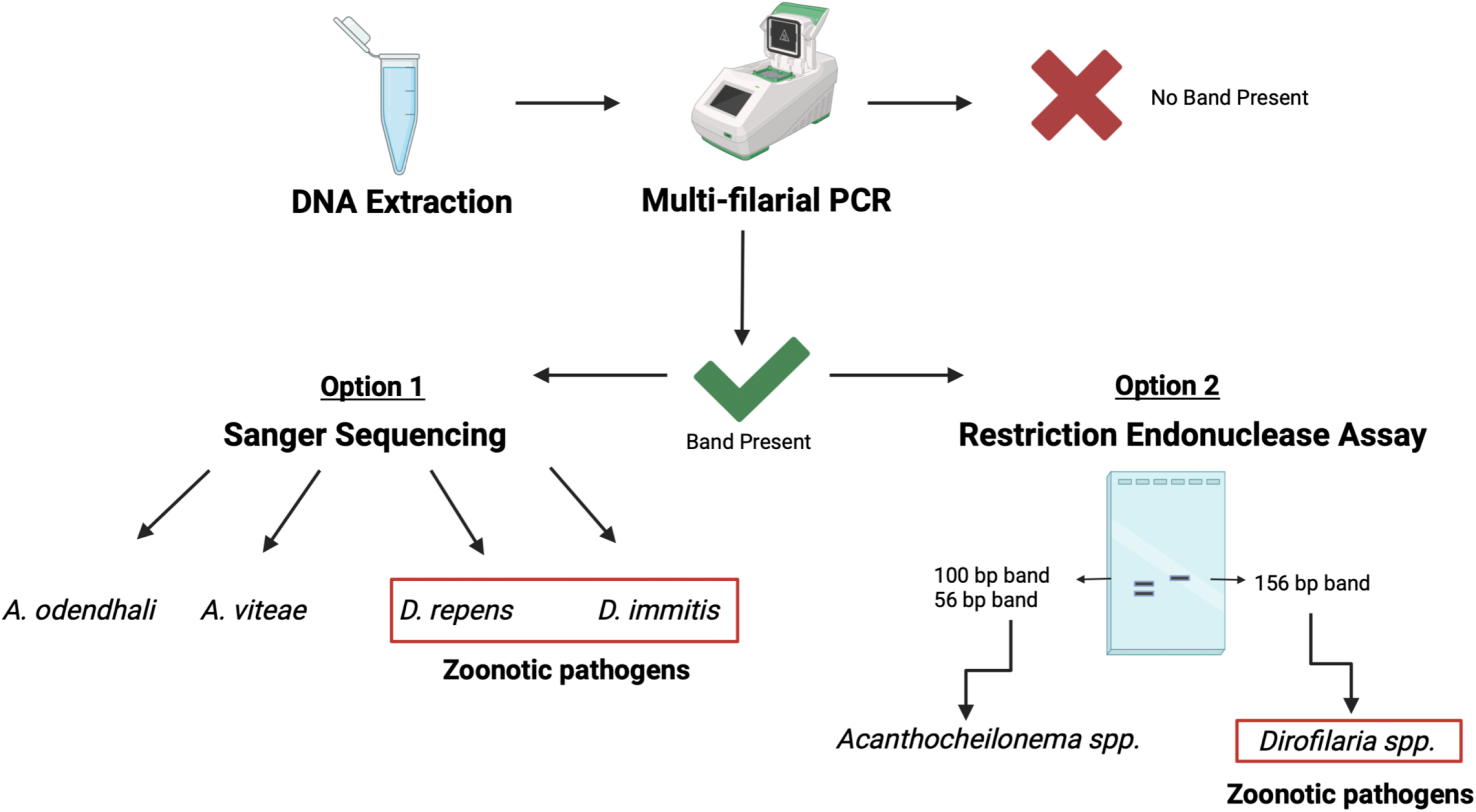
Proposed workflow for identification of DNA from filarial nematode species in GSL blood samples. Figure created with BioRender.com.

The PCR results of the multifilarial assay for all 28 GSLs showed three blood samples (10.7%) with single amplicons of the correct size (156 bp) (G9, G16, G17) (Figure 3A), including the sample (G16) that previously tested positive for circulating *D. immitis* antigens. The BLAST and phylogenetic analyses performed with amplicons from Sanger sequencing revealed that the PCR amplicon from the antigen‐positive sea lion (G16) matched that of *D. immitis* (percent identity 100%). One GSL blood sample (G9) possessed DNA from *D. immitis* (percent identity 100%) despite not testing positive for heartworm antigens. The remaining sample (G17) matched *D. repens* (percent identity 100%), which represents the first reported detection of DNA from this filarial nematode in a GSL (Figure 3B). All alignments for the positive GSL samples can be found in Appendix S3. The melt curve profiles generated from the qPCR adaptation of our multifilarial PCR for G9, G16, and G17 were 82°C, 82.0°C, and 83°C, respectively, consistent with the profiles of the control samples for the filarial species identified by Sanger sequencing (Table 3). The TatI restriction endonuclease products were consistent with the *in silico* predicted results and produced two bands of approximately 56 and 100 bp when the 156 bp amplicon of the *Acanthocheilonema* species was digested (Figure 5A,B). For the controls for both *Dirofilaria* species and the three positive GSLs, the 156 bp amplicon was not cut by TatI (Figure 5B). Using the cumulative results of our novel workflow (Figure 6), we report that *D. immitis* had a prevalence of 7.1% in our dataset while *D. repens* had a prevalence of 3.6%.

## 4 Discussion

This study describes the development of a molecular test for the fast and accurate identification of DNA from nematode species including *D. immitis* , *D. repens* , *A. viteae*, and *A. odendhali* in the GSL. Our previous study demonstrated that GSL is susceptible to *D. immitis* infection (Gregory et al. 2023), and our work is the first to use molecular tools to determine the prevalence of heartworms and report the presence of *D. repens* in this species. This molecular test is a valuable tool for monitoring and understanding the impact of *D. immitis* and related filarial worms on the GSL, and for identifying epidemiologic factors that could be leveraged to minimize transmission from reservoir species such as the dog.

Current commercial antigen tests for *D. immitis* detect glycoproteins found in the reproductive tract of female worms and are therefore unsuitable for the detection of immature, low‐worm burden, and all‐male infections (Starkey et al. 2017; Venco et al. 2017). Various filarial species cross‐react with commercial tests (Krucik, Van Bonn, and Johnson 2016), necessitating reliable species‐specific identification. As our assay targets the genomic DNA of the pathogen, it provides a reliable means for the detection of *D. immitis* regardless of sex and life stage, as evidenced by the successful detection of DNA from microfilaria and adult specimens of both sexes.

The sensitivity of our PCR assay appears to be greater than that of commercially available antigen test kits as it detected *D. immitis* DNA in one GSL that tested negative for antigens and microfilariae, at a copy number significantly lower than those documented in active canine infections (1000 mf/μL) (McTier et al. 2017). The results of the qPCR assay suggest that detection of as little as one parasite is possible, which offers the potential to detect the presence of these pathogens before infections can fully develop and allows for timely intervention. Cumulatively, these results demonstrate the efficacy of our molecular tools for the detection of nematode DNA in the GSL.

Three GSLs were PCR‐positive for the presence of nematode DNA, one of which was also positive for circulating *D. immitis* antigens. One GSL that tested positive for *D. immitis* antigens did not test positive for DNA. Three antigen tests were performed for this individual, and two of three of the tests had visible, albeit faint signals. Given the inconsistencies in the antigen results, the sensitivity of the molecular test, and the documented lack of specificity for the antigen test kits in related wild species (Alho et al. 2017; Krucik, Van Bonn, and Johnson 2016; Venco et al. 2017), the result of the antigen test could have been a false positive.

The primers selected for the multifilarial assay were based on two main criteria: the conservation of primer sequences across different species of filarial nematodes and the presence of unique species‐specific sequences within the central portion of the amplicon. Consequently, only species with available complete 28S sequences were used to create the synthetic controls used in this study. Despite this, the high degree of similarity in the primer‐binding regions among the targeted species, as well as other less related species indicates that these primers might also be effective for other species whose gene sequences have not been published. A common phocid nematode species, *A. spirocauda* could not be included as no complete 28S sequences were available. Although it may be a species of interest, it primarily infects phocid species, so it is unlikely to be the cause of the positive antigen test in the sea lion that did not have detectable DNA for the four species tested.

The primers used for our multifilarial PCR have been successfully adapted to qPCR, which can quantify parasite burden. However, this method requires more expensive equipment. Despite not providing quantitative data, traditional endpoint PCR can be performed with basic laboratory equipment and provides an accessible means for infectious disease exploration. A similar workflow, PCR followed by sequencing of amplified DNA and identification of species through BLAST and phylogenetic analysis, is routinely used in clinical settings (Dark, Dean, and Warhurst 2009; Zucol et al. 2006). The use of restriction endonucleases offers a suitable alternative for identifying filarial nematodes present with minimal equipment. We used the TatI restriction enzyme to rapidly differentiate DNA from the genera *Dirofilaria* and *Acanthocheilonema*. *Dirofilaria* spp. are responsible for dirofilariasis and are of significant public health concern as they are known to cause disease in a wide range of species, including humans. In contrast, infections caused by *Acanthocheilonema* spp. are generally not zoonotic. As discussed in a study of *D. repens* in southern Finland, most filarioid nematodes that parasitize animals can infect humans and develop to infective stages under suitable conditions (Pietikäinen et al. 2017). Thus, genus‐level identification is valuable for informing ongoing surveillance of companion animal pathogens in the GSL and surveillance of zoonotic pathogens under the scope of the Global One Health approach (Figure 6).

While our PCR‐based approach successfully identified the presence of filarial nematode‐specific DNA in GSL samples, a notable limitation of this study was the inability to corroborate these findings with direct observation of live worms in the blood due to logistical constraints of field sampling. The absence of such validation means we cannot definitively confirm the current infection status based on PCR results alone. Future efforts will aim to integrate parasitological validation by obtaining and examining biological samples when possible. This would not only confirm the presence of live heartworms but also enhance our understanding of the parasite's life cycle in wild hosts. Despite this limitation, the PCR assay we have developed offers a valuable and sensitive tool for the detection and identification of filarial nematodes in wild pinnipeds. This approach is particularly beneficial in wildlife populations where traditional diagnostic methods may be challenging to implement. This assay will enable ongoing surveillance and has the potential to contribute significantly to conservation efforts by informing management strategies for the health of GSL populations. Moving forward, the addition of more publicly available genetic data for nematode species, and the integration of PCR with direct parasitological observations, will strengthen the reliability of our molecular approach and provide a more comprehensive understanding of heartworm disease dynamics in these pinnipeds.

Our current understanding of the relationship between companion animal diseases and the health of GSL is limited (Gregory et al. 2023). However, the findings from this study provide further evidence that GSL populations are susceptible to infection from companion animal pathogens. As there are effective prevention and management measures for *D. immitis* infections for dogs, understanding the role dogs play in the transmission of infections to GSL is critical (Gregory et al. 2023). Although no clinical signs of infection were observed in the PCR (or antigen) positive individuals at the time of sampling, the large number of adult worms found in the necropsied GSL is evidence of the potential for infection in this species to cause disease. In GSLs, even subclinical infections may interfere with oxygenation during physical exertion, which could increase the risk of predation (Gregory et al. 2023). These detrimental effects could be exacerbated if the worm burden is significant or in periods of nutritional stress such as El Niño events. Thus, infection by *Dirofilaria* spp. could be detrimental to the survivability of the GSL (Páez‐Rosas et al. 2021).

The conservation of Galapagos species hinges on the success of efforts to manage the interplay between humans, domestic animals, and the wildlife and ecosystems of the archipelago (Padilla et al. 2018; Galapagos Conservation Trust). To monitor and control nematode infections, fast and accurate identification of causative species is imperative (Gioia et al. 2010). Therefore, the development of rapid and accessible molecular‐based detection methods is critical for monitoring and mitigating infections in native wildlife populations (Oh, Kim, and Sung 2017; Gioia et al. 2010).

## 5 Conclusions

The molecular methods developed in this study represent an improvement in the detection and identification of filariasis in wild pinnipeds and can be used to complement traditional diagnostic methods. The proposed workflow (Figure 6) facilitates the rapid and efficient identification of nematode DNA. The PCR primers designed for this assay can be used in a variety of host species for reliable detection of filarial nematode DNA. Although our study was limited, our assay provides a valuable tool for the needed surveillance of filarial infections in the GSL. The findings of our study suggest that further research on the epidemiology of filarial nematodes in Galapagos sea lions is warranted to better assess the species' susceptibility to infection and their role as potential reservoirs.

## Author Contributions

**Isabella G. Livingston:** conceptualization (equal), data curation (lead), formal analysis (lead), investigation (lead), methodology (lead), project administration (equal), visualization (lead), writing – original draft (lead), writing – review and editing (equal). **Taylor M. Gregory:** conceptualization (equal), investigation (supporting), project administration (equal), writing – review and editing (equal). **Eleanor C. Hawkins:** conceptualization (equal), investigation (supporting), project administration (equal), writing – review and editing (equal). **Ashley Cave:** conceptualization (supporting), investigation (supporting), writing – review and editing (equal). **Andrea Loyola:** resources (supporting), writing – review and editing (equal). **Shelly L. Vaden:** conceptualization (supporting), project administration (equal), writing – review and editing (equal). **Diane Deresienski:** conceptualization (equal), project administration (supporting), writing – review and editing (equal). **Marjorie Riofrío‐Lazo:** project administration (equal), resources (equal), writing – review and editing (equal). **Gregory A. Lewbart:** conceptualization (equal), funding acquisition (equal), project administration (equal), writing – review and editing (equal). **Diego Páez‐Rosas:** project administration (equal), resources (equal), supervision (equal), writing – review and editing (equal). **Matthew Breen:** conceptualization (equal), funding acquisition (equal), methodology (supporting), project administration (equal), resources (equal), supervision (equal), visualization (supporting), writing – original draft (supporting), writing – review and editing (equal).

## Conflicts of Interest

The authors declare no conflicts of interest.

## Supporting information


Appendix S1.

Appendix S2.

Appendix S3.

## Data Availability

All protocols used and data generated from this study are detailed in the text or associated figures. Sequencing data and alignments can be found in Appendix S3. Melt curve profiles for the qPCR assay are shown in Appendix S2. Sample metadata and [Link], [Link], [Link] can be found in the Dryad repository doi:10.5061/dryad.v6wwpzh5g.
