# Supplementary material for: Molecular Discovery of Filarial Nematode DNA in an Endangered Wild Pinniped (Galapagos Sea Lion, Zalophus wollebaeki)
Source: Ecol Evol. 2024 Nov 25;14(11):e70596. doi: 10.1002/ece3.70596 (PMC11586683; doi:10.1002/ece3.70596)
Supplement: Supplementary file 1 — Appendix S1. [file ECE3-14-e70596-s001.docx]

**Appendix A**

28S amplicon sequences for various filarial nematode species. Green text denotes the designed primer sequences. The red text denotes species specific base differences.

**D.REPENS 28S**

| 5’-CAGTCCATAGAAGGTGCTAGACCTGTACGGGTGAAAAAAGTTATGGTAGATTATACTTTGGAGTCTTGTTTTGTTTGAGATTGCAGCCCAAAGAGGGTGGTAA ACCTCATCTAAGGCTAAATACGACCACGAGACCGATAGCAAACAAGTACCGTGAGG-3’ |
| --- |

**D.IMMITIS 28S**

| 5’-CAGTCCATAGAAGGTGCTAGACCTGTACGGGTGAAAAAAGTTACGATATGTTATACTTTGGAGTCGGGTTGTTTGAGATTGCAGCCCAAAGAGGGTGGTAA ACCTCATCTAAGGCTAAATATGACCACGAGACCGATAGCAAACAAGTACCGTGAGG-3’ |
| --- |

**A.VITEAE 28S**

| 5’-CAGTCCATAGAAGGTGCTAGACCTGTACGGGTGAGAAGAGTTACGATAGATTGTACTTTGGAGTCGGGTTGTTTGAGATTGCAGCCCAAAGAGGGTGGTAA ACCTCATCTAAGGCTAAATACGACCACGAGACCGATAGCAAACAAGTACCGTGAGG-3’ |
| --- |

**A.ODENDHALI 28S**

5’-CAGTCCATAGAAGGTGCTAGACCTGTACGGGTGAGGAAAGTTACGATAGATTGTACTCTGGAGTCGGGTTGTTTGAGATTGCAGCCCAAAGAGGGTGGTAA ACCTCATCTAAGGCTAAATACGACCACGAGACCGATAGCAAACAAGTACCGTGAG-3’

**D.GRACILE 28S**

| 5’-CAGTCCATAGAAGGTGCTAGACCTGTACGGGTGAAAAAAGTTACGGTAGGTTATTCCTTGGAGTCGGGTTGTTTGAGATTGCAGCCCAAAGAGGGTGGTAA ACCTCATCTAAGGCTAAATACGACCACGAGACCGATAGTAAACAAGTACCGTGAGG-3’ |
| --- |

**B.MALAYI 28S**

| 5’-CAGTCCATAGAAGGTGCTAGACCTGTACGGGTGAAAAAAGTTACGGTAGGTTATTCCTTGGAGTCGGGTTGTTTGAGATTGCAGCCCAAAGAGGGTGGTAA ACCTCATCTAAGGCTAAATACGACCATGAGACCGATAGCAAACAAGTACCGTGAGG-3’  **D.ROBINI 28S**  5’-CAGTCCATAGAAGGTGCTAGACCTGTACGGGTGAAAAAAGTTACGGTAGGTTATTCCTTGGAGTCGGGTTGTTTGAGATTGCAGCCCAAAGAGGGTGGTAA ACCTCATCTAAGGCTAAATACGACCACGAGACCGATAGCAAACAAGTACCGTGAGG-3’ |
| --- |
