# Supplementary material for: Molecular Discovery of Filarial Nematode DNA in an Endangered Wild Pinniped (Galapagos Sea Lion, Zalophus wollebaeki)
Source: Ecol Evol. 2024 Nov 25;14(11):e70596. doi: 10.1002/ece3.70596 (PMC11586683; doi:10.1002/ece3.70596)
Supplement: Supplementary file 2 — Appendix S2. [file ECE3-14-e70596-s003.docx]

Appendix B

Appendix B shows melt curve profiles for each of the filarial species tested and examples of melt curve profiles for samples negative for filarial nematode DNA.


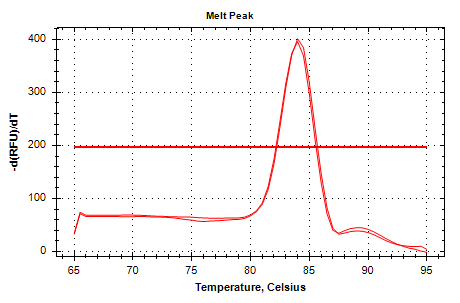

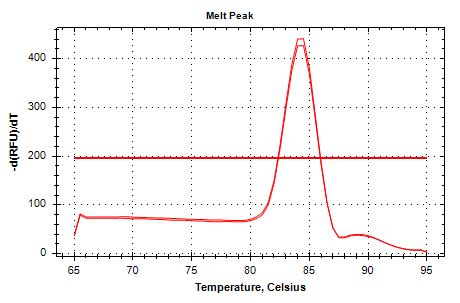


AVE Melt Curve

AOD Melt Curve


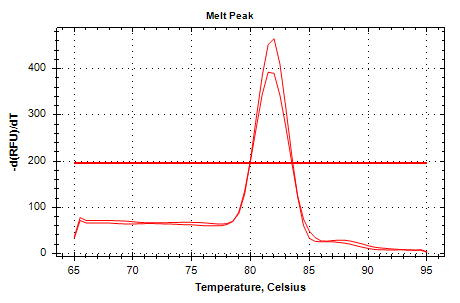

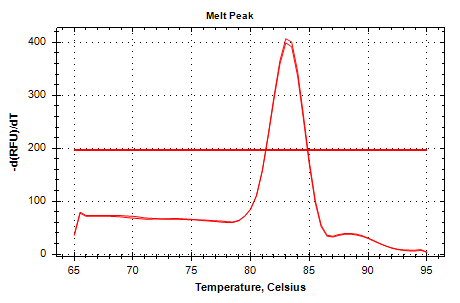


DIM Melt Curve

DRE Melt Curve


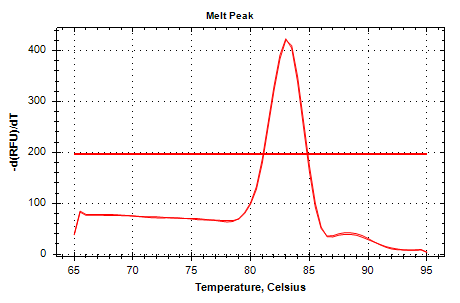

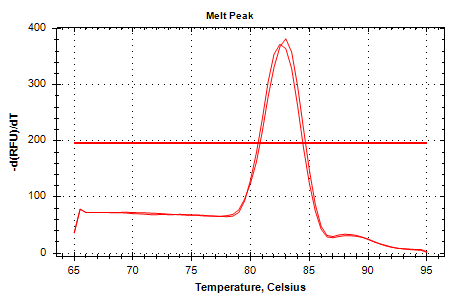


DRE + DIM Melt Curve


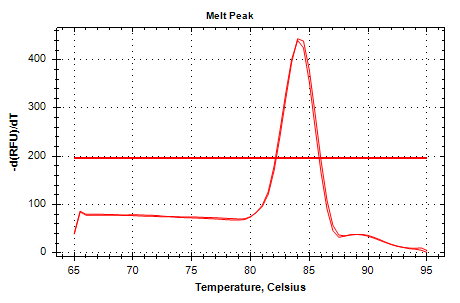

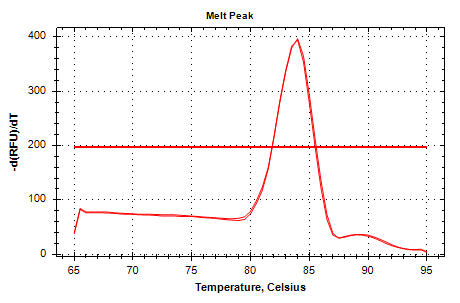


AOD + DIM Melt Curve


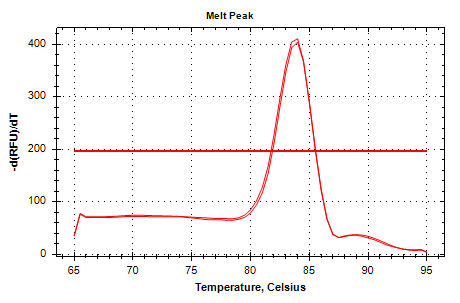

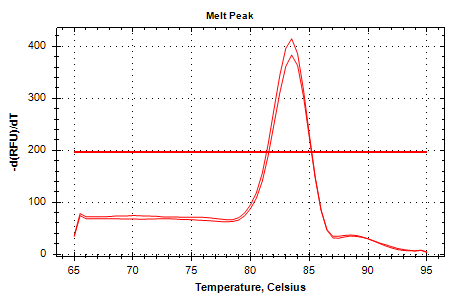


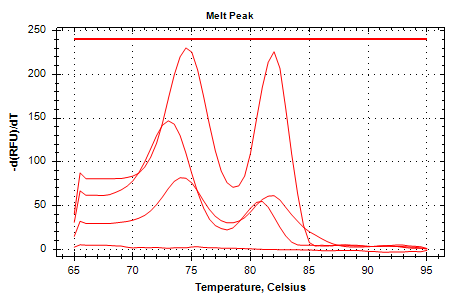

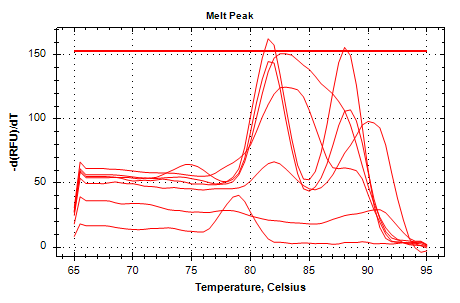

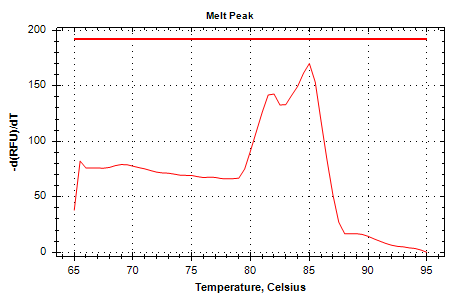

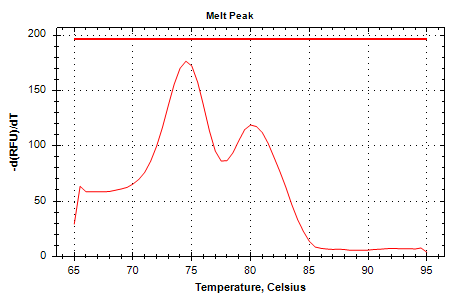


Examples of Negative Curves

AVE + DRE Melt Curve
