# Supplementary material for: Molecular Discovery of Filarial Nematode DNA in an Endangered Wild Pinniped (Galapagos Sea Lion, Zalophus wollebaeki)
Source: Ecol Evol. 2024 Nov 25;14(11):e70596. doi: 10.1002/ece3.70596 (PMC11586683; doi:10.1002/ece3.70596)
Supplement: Supplementary file 3 — Appendix S3. [file ECE3-14-e70596-s002.pdf]

Appendix C

Appendix C shows the text alignments for all alignments performed using CLUSTAL. Results are shown for each of the three positive GSL samples compared to the expected amplicon sequences and control sample amplicon sequences for each filarial species.

```
CLUSTAL O(1.2.4) multiple sequence alignment


A.odend_synthetic      CCAGTCCATAGAAGGTGCTAGACCTGTACGGGTGAGGAAAGTTACGATAGATTGTACTCT     60
A.odendhali_28S        CCAGTCCATAGAAGGTGCTAGACCTGTACGGGTGAGGAAAGTTACGATAGATTGTACTCT     60
A.viteae_28S           CCAGTCCATAGAAGGTGCTAGACCTGTACGGGTGAGAAGAGTTACGATAGATTGTACTTT     60
A.vit_synthetic        ------CATAGAAGGTGCTAGACCTGTACGGGTGAGAAGAGTTACGATAGATTGTACTTT     54
D.rep_synthetic        ----TCCATAGAAGGTGCTAGACCTGTACGGGTGAAAAAAGTTATGGTAGATTATACTTT     56
D.repens_28S           -CAGTCCATAGAAGGTGCTAGACCTGTACGGGTGAAAAAAGTTATGGTAGATTATACTTT     59
D.imm_synthetic        ------------------------------GGTGAAAAAAGTTACGATATGTTATACTTT     30
D.immitis_28S          -CAGTCCATAGAAGGTGCTAGACCTGTACGGGTGAAAAAAGTTACGATATGTTATACTTT     59
G9_amplicon            CTAGTCCATAGAAGGTGCTAGACCTGTACGGGTGAAAAAAGTTACGATATGTTATACTTT     60
                                                     *****  * ***** * **  ** **** *

A.odend_synthetic      GGAGTCGGGTTGTTTGAGATTGCAGCCCAAAGAGGGTGGTAAACCTCATCTAAGGCTAAA     120
A.odendhali_28S        GGAGTCGGGTTGTTTGAGATTGCAGCCCAAAGAGGGTGGTAAACCTCATCTAAGGCTAAA     120
A.viteae_28S           GGAGTCGGGTTGTTTGAGATTGCAGCCCAAAGAGGGTGGTAAACCTCATCTAAGGCTAAA     120
A.vit_synthetic        GGAGTCGGGTTGTTTGAGATTGCAGCCCAAAGAGGGTGGTAAACCTCATCTAAGGCTAAA     114
D.rep_synthetic        GGAGTCGGGTTGTTTGAGATTGCAGCCCAAAGAGGGTGGTAAACCTCATCTAAGGCTAAA     116
D.repens_28S           GGAGTCGGGTTGTTTGAGATTGCAGCCCAAAGAGGGTGGTAAACCTCATCTAAGGCTAAA     119
D.imm_synthetic        GGAGTCGGGTTGTTTGAGATTGCAGCCCAAAGAGGGTGGTAAACCTCATCTAAGGCTAAA     90
D.immitis_28S          GGAGTCGGGTTGTTTGAGATTGCAGCCCAAAGAGGGTGGTAAACCTCATCTAAGGCTAAA     119
G9_amplicon            GGAGTCGGGTTGTTTGAGATTGCAGCCCAAAGAGGGTGGTAAACCTCATCTAAGGCTAAA     120
                       ************************************************************

A.odend_synthetic      TACGACCACGAGACCGATAGCAAACAAGTACCGTGAG--- 157
A.odendhali_28S        TACGACCACGAGACCGATAGCAAACAAGTACCGTGAG--- 157
A.viteae_28S           TACGACCACGAGACCGATAGCAAACAAGTACCGTGAG--- 157
A.vit_synthetic        TACGACCACGAGACCGATAGCAAACAAGTACCGTGAG--- 151
D.rep_synthetic        TACGACCACGAGACCGATAGCAAACAAGTACCGTGAG--- 153
D.repens_28S           TACGACCACGAGACCGATAGCAAACAAGTACCGTGAG--- 156
D.imm_synthetic        TATGACCACGAGACCGATAGCAAACAAGTACCGTGAGAAT 130
D.immitis_28S          TATGACCACGAGACCGATAGCAAACAAGTACCGTGAG--- 156
G9_amplicon            TATGACCACGAGACCGATAGCAAACAAGTACCGTGAGA-- 158
                       ** **********************************
```

CLUSTAL O(1.2.4) multiple sequence alignment

```
Aodend          CCAGTCCATAGAAGGTGCTAGACCTGTACGGGTGAGGAAAGTTACGATAGATTGTACTCT   60
A.odendhali     CCAGTCCATAGAAGGTGCTAGACCTGTACGGGTGAGGAAAGTTACGATAGATTGTACTCT   60
A.vit           ------CATAGAAGGTGCTAGACCTGTACGGGTGAGAAGAGTTACGATAGATTGTACTTT   54
A.viteae        CCAGTCCATAGAAGGTGCTAGACCTGTACGGGTGAGAAGAGTTACGATAGATTGTACTTT   60
D.rep           ----TCCATAGAAGGTGCTAGACCTGTACGGGTGAAAAAAGTTATGGTAGATTATACTTT   56
D.repens        -CAGTCCATAGAAGGTGCTAGACCTGTACGGGTGAAAAAAGTTATGGTAGATTATACTTT   59
D.imm           ------------------------------GGTGAAAAAAGTTACGATATGTTATACTTT   30
G16             CCAGTCCATAGAAGGTGCTAGACCTGTACGGGTGAAAAAAGTTACGATATGTTATACTTT   60
D.immitis       -CAGTCCATAGAAGGTGCTAGACCTGTACGGGTGAAAAAAGTTACGATATGTTATACTTT   59
                                              *****  * ***** * **  ** **** *

Aodend          GGAGTCGGGTTGTTTGAGATTGCAGCCCAAAGAGGGTGGTAAACCTCATCTAAGGCTAAA   120
A.odendhali     GGAGTCGGGTTGTTTGAGATTGCAGCCCAAAGAGGGTGGTAAACCTCATCTAAGGCTAAA   120
A.vit           GGAGTCGGGTTGTTTGAGATTGCAGCCCAAAGAGGGTGGTAAACCTCATCTAAGGCTAAA   114
A.viteae        GGAGTCGGGTTGTTTGAGATTGCAGCCCAAAGAGGGTGGTAAACCTCATCTAAGGCTAAA   120
D.rep           GGAGTCGGGTTGTTTGAGATTGCAGCCCAAAGAGGGTGGTAAACCTCATCTAAGGCTAAA   116
D.repens        GGAGTCGGGTTGTTTGAGATTGCAGCCCAAAGAGGGTGGTAAACCTCATCTAAGGCTAAA   119
D.imm           GGAGTCGGGTTGTTTGAGATTGCAGCCCAAAGAGGGTGGTAAACCTCATCTAAGGCTAAA   90
G16             GGAGTCGGGTTGTTTGAGATTGCAGCCCAAAGAGGGTGGTAAACCTCATCTAAGGCTAAA   120
D.immitis       GGAGTCGGGTTGTTTGAGATTGCAGCCCAAAGAGGGTGGTAAACCTCATCTAAGGCTAAA   119
                ************************************************************

Aodend          TACGACCACGAGACCGATAGCAAACAAGTACCGTGAG---      157
A.odendhali     TACGACCACGAGACCGATAGCAAACAAGTACCGTGAG---      157
A.vit           TACGACCACGAGACCGATAGCAAACAAGTACCGTGAG---      151
A.viteae        TACGACCACGAGACCGATAGCAAACAAGTACCGTGAG---      157
D.rep           TACGACCACGAGACCGATAGCAAACAAGTACCGTGAG---      153
D.repens        TACGACCACGAGACCGATAGCAAACAAGTACCGTGAG---      156
D.imm           TATGACCACGAGACCGATAGCAAACAAGTACCGTGAGAAT      130
G16             TATGACCACGAGACCGATAGCAAACAAGTACCGTGAGA--      158
D.immitis       TATGACCACGAGACCGATAGCAAACAAGTACCGTGAG---      156
                ** **********************************
```

```
CLUSTAL O(1.2.4) multiple sequence alignment


A.odend_synthetic      CCAGTCCATAGAAGGTGCTAGACCTGTACGGGTGAGGAAAGTTACGATAGATTGTACTCT     60
A.odendhali_28S        CCAGTCCATAGAAGGTGCTAGACCTGTACGGGTGAGGAAAGTTACGATAGATTGTACTCT     60
A.viteae_28S           CCAGTCCATAGAAGGTGCTAGACCTGTACGGGTGAGAAGAGTTACGATAGATTGTACTTT     60
A.vit_synthetic        ------CATAGAAGGTGCTAGACCTGTACGGGTGAGAAGAGTTACGATAGATTGTACTTT     54
D.rep_synthetic        ----TCCATAGAAGGTGCTAGACCTGTACGGGTGAAAAAAGTTATGGTAGATTATACTTT     56
D.repens_28S           -CAGTCCATAGAAGGTGCTAGACCTGTACGGGTGAAAAAAGTTATGGTAGATTATACTTT     59
G17_amplicon           -CAGTCCATAGAAGGTGCTAGACCTGTACGGGTGAAAAAAGTTATGGTAGATTATACTTT     59
D.immitis_28S          -CAGTCCATAGAAGGTGCTAGACCTGTACGGGTGAAAAAAGTTACGATATGTTATACTTT     59
D.imm_synthetic        ------------------------------GGTGAAAAAAGTTACGATATGTTATACTTT     30
                                                     *****  * ***** * **  ** **** *

A.odend_synthetic      GGAGTCGGGTTGTTTGAGATTGCAGCCCAAAGAGGGTGGTAAACCTCATCTAAGGCTAAA     120
A.odendhali_28S        GGAGTCGGGTTGTTTGAGATTGCAGCCCAAAGAGGGTGGTAAACCTCATCTAAGGCTAAA     120
A.viteae_28S           GGAGTCGGGTTGTTTGAGATTGCAGCCCAAAGAGGGTGGTAAACCTCATCTAAGGCTAAA     120
A.vit_synthetic        GGAGTCGGGTTGTTTGAGATTGCAGCCCAAAGAGGGTGGTAAACCTCATCTAAGGCTAAA     114
D.rep_synthetic        GGAGTCGGGTTGTTTGAGATTGCAGCCCAAAGAGGGTGGTAAACCTCATCTAAGGCTAAA     116
D.repens_28S           GGAGTCGGGTTGTTTGAGATTGCAGCCCAAAGAGGGTGGTAAACCTCATCTAAGGCTAAA     119
G17_amplicon           GGAGTCGGGTTGTTTGAGATTGCAGCCCAAAGAGGGTGGTAAACCTCATCTAAGGCTAAA     119
D.immitis_28S          GGAGTCGGGTTGTTTGAGATTGCAGCCCAAAGAGGGTGGTAAACCTCATCTAAGGCTAAA     119
D.imm_synthetic        GGAGTCGGGTTGTTTGAGATTGCAGCCCAAAGAGGGTGGTAAACCTCATCTAAGGCTAAA     90
                       ************************************************************

A.odend_synthetic      TACGACCACGAGACCGATAGCAAACAAGTACCGTGAG--- 157
A.odendhali_28S        TACGACCACGAGACCGATAGCAAACAAGTACCGTGAG--- 157
A.viteae_28S           TACGACCACGAGACCGATAGCAAACAAGTACCGTGAG--- 157
A.vit_synthetic        TACGACCACGAGACCGATAGCAAACAAGTACCGTGAG--- 151
D.rep_synthetic        TACGACCACGAGACCGATAGCAAACAAGTACCGTGAG--- 153
D.repens_28S           TACGACCACGAGACCGATAGCAAACAAGTACCGTGAG--- 156
G17_amplicon           TACGACCACGAGACCGATAGCAAACAAGTACCGTGAG--- 156
D.immitis_28S          TATGACCACGAGACCGATAGCAAACAAGTACCGTGAG--- 156
D.imm_synthetic        TATGACCACGAGACCGATAGCAAACAAGTACCGTGAGAAT 130
                       ** **********************************
```
